# Supplementary material for: Glycogen phase-separation drives macromolecular rearrangement and asymmetric division in E. coli
Source: EMBO J. 2025 Nov 3;44(24):7434–76. doi: 10.1038/s44318-025-00621-y (PMC12706056; doi:10.1038/s44318-025-00621-y)
Supplement: Supplementary file 1 — Table EV1 [file 44318_2025_621_MOESM1_ESM.docx]

**Table EV1. Calculated concentrations of glycogen and proteins in *E. coli* under different growth conditions.**

| **Glycogen** | | | |
| --- | --- | --- | --- |
| Reported (mg/g dry weight) | Conversion (g/L) | Conditions | References |
| 13 | 1.43 | Exponential phase, 1% glucose in enriched medium (with yeast extract) at 37°C | (Govons et al., 1969; Preiss & Romeo, 1994) |
| 2.5 | 8.25 | Exponential phase, glucose minimal media at 37°C, 40 min division rate | (Ingraham et al., 1983) |
| 2.8-4.4 | 9.24-14.52 | Stationary phase, LB at 37°C | (Wang et al., 2019) |
| 19 | 20.9 | Stationary phase, 1% glucose in enriched medium (with yeast extract) at 37°C | (Govons et al., 1969; Preiss & Romeo, 1994) |
| **Proteins** | | | |
| Reported | Conversion (mM) | Conditions | References |
| 2.35x10^6^ proteins per cell | 4.48 | Exponential phase, glucose minimal medium at 37°C, 40 min division rate | (Neidhardt & Curtiss, 1996) |
| 0.24 g/mL | 9.6-12 | Calculated for proteins between 20 and 25 kDa | (Ingraham et al., 1983) |

Glycogen and protein concentrations used in our study were derived from reported values for *E. coli* cells growing under different conditions. Glycogen concentrations from dry weight measurements (mg/g cells) were converted to wet weight values using a water content value of 70% (Bremer & Dennis, 2008; Feijó Delgado et al., 2013). To obtain the concentrations in g/L, we used a cell density of 1.1 g/mL (Kubitschek et al., 1983; Loferer-Krössbacher et al., 1998). To calculate the protein concentrations in mM from proteins per cell, we used a cell volume of 0.86 µm^3^ (Kubitschek & Friske, 1986; Milo, 2013). To obtain the mM concentrations from g/mL, we used the indicated molecular weight range.

**References**

Bremer, H., & Dennis, P. P. (2008). Modulation of chemical composition and other parameters of the cell at different exponential growth rates. *EcoSal Plus*, *3*(1). https://doi.org/10.1128/ecosal.5.2.3

Feijó Delgado, F., Cermak, N., Hecht, V. C., Son, S., Li, Y., Knudsen, S. M., Olcum, S., Higgins, J. M., Chen, J., Grover, W. H., & Manalis, S. R. (2013). Intracellular water exchange for measuring the dry mass, water mass and changes in chemical composition of living cells. *PloS One*, *8*(7), e67590. https://doi.org/10.1371/journal.pone.0067590

Govons, S., Vinopal, R., Ingraham, J., & Preiss, J. (1969). Isolation of mutants of Escherichia coli B altered in their ability to synthesize glycogen. *Journal of Bacteriology*, *97*(2), 970–972. https://doi.org/10.1128/jb.97.2.970-972.1969

Ingraham, J. L., Maaløe, O., & Neidhardt, F. C. (1983). *Growth of the bacterial cell*. Sunderland (Mass.) : Sinauer associates.

Kubitschek, H. E., Baldwin, W. W., & Graetzer, R. (1983). Buoyant density constancy during the cell cycle of Escherichia coli. *Journal of Bacteriology*, *155*(3), 1027–1032. https://doi.org/10.1128/jb.155.3.1027-1032.1983

Kubitschek, H. E., & Friske, J. A. (1986). Determination of bacterial cell volume with the Coulter Counter. *Journal of Bacteriology*, *168*(3), 1466–1467. https://doi.org/10.1128/jb.168.3.1466-1467.1986

Loferer-Krössbacher, M., Klima, J., & Psenner, R. (1998). Determination of bacterial cell dry mass by transmission electron microscopy and densitometric image analysis. *Applied and Environmental Microbiology*, *64*(2), 688–694. https://doi.org/10.1128/AEM.64.2.688-694.1998

Milo, R. (2013). What is the total number of protein molecules per cell volume? A call to rethink some published values. *BioEssays*, *35*(12), 1050–1055. https://doi.org/10.1002/bies.201300066

Neidhardt, F. C., & Roy Curtiss. (1996). Escherichia coli and Salmonella : cellular and molecular biology. In *Journal of Clinical Pathology* (2nd ed., Issue 5). ASM Press. https://jcp.bmj.com/lookup/doi/10.1136/jcp.39.5.503

Preiss, J., & Romeo, T. (1994). Molecular biology and regulatory aspects of glycogen biosynthesis in bacteria. *Progress in Nucleic Acid Research and Molecular Biology*, *47*(C), 299–329. https://doi.org/10.1016/s0079-6603(08)60255-x

Wang, L., Liu, Q., Tan, X., Wang, Z., Wang, M., Wise, M. J., Li, C., Ma, C., Li, E., Deng, B., Du, Y., Tang, D., & Gilbert, R. G. (2019). Molecular structure of glycogen in Escherichia coli. *Biomacromolecules*, *20*(7), 2821–2829. https://doi.org/10.1021/acs.biomac.9b00586
