# Supplementary material for: Glycogen phase-separation drives macromolecular rearrangement and asymmetric division in E. coli
Source: EMBO J. 2025 Nov 3;44(24):7434–76. doi: 10.1038/s44318-025-00621-y (PMC12706056; doi:10.1038/s44318-025-00621-y)
Supplement: Supplementary file 2 — Table EV2 [file 44318_2025_621_MOESM2_ESM.docx]

**Table EV2. Calculated concentrations of ribosomes and polysomes used in this study.**

| **Ribosomesper cell** | **Conditions** | **Cell volume (µm^3^)** | **Estimated ribosome concentration (µM)** | **Estimated polysome concentration (µM)** | **References** |
| --- | --- | --- | --- | --- | --- |
| 3500 | Stationary phase, LB at 37°C | 1 | 5.8 | 1.16 | (Nilsson et al., 1997) |
| 15000 | Exponential phase, M9gly at 37°C, | 1 | 24.9 | 4.98 | (Bremer & Dennis, 2008) |
|  | 60 min doubling time |  |  |  |  |
| 61000 | Exponential phase, M9gluCAA at 37°C, | 3.5 | 28.9 | 5.79 | (Bremer & Dennis, 2008) |
|  | 24 min doubling time |  |  |  |  |

Concentrations were determined from the reported numbers of ribosomes per cell using the Avogadro constant to calculate the number of moles per cell, then dividing by the reported cell volume values indicated (Floc’h et al., 2019; Kubitschek & Friske, 1986). For polysomes, an active fraction of 0.8 percent was used (Bremer & Dennis, 2008) and a polysome was assumed to consist of four ribosomes (Brandt et al., 2009).

**References**

Brandt, F., Etchells, S. A., Ortiz, J. O., Elcock, A. H., Hartl, F. U., & Baumeister, W. (2009). The native 3D organization of bacterial polysomes. *Cell*, *136*(2), 261–271. https://doi.org/10.1016/j.cell.2008.11.016

Bremer, H., & Dennis, P. P. (2008). Modulation of chemical composition and other parameters of the cell at different exponential growth rates. *EcoSal Plus*, *3*(1). https://doi.org/10.1128/ecosal.5.2.3

Floc’h, K., Lacroix, F., Servant, P., Wong, Y.-S., Kleman, J.-P., Bourgeois, D., & Timmins, J. (2019). Cell morphology and nucleoid dynamics in dividing Deinococcus radiodurans. *Nature Communications*, *10*(1), 3815. https://doi.org/10.1038/s41467-019-11725-5

Kubitschek, H. E., & Friske, J. A. (1986). Determination of bacterial cell volume with the Coulter Counter. *Journal of Bacteriology*, *168*(3), 1466–1467. https://doi.org/10.1128/jb.168.3.1466-1467.1986

Nilsson, M., Bülow, L., & Wahlund, K. G. (1997). Use of flow field-flow fractionation for the rapid quantitation of ribosome and ribosomal subunits in Escherichia coli at different protein production conditions. *Biotechnology and Bioengineering*, *54*(5), 461–467. https://doi.org/10.1002/(SICI)1097-0290(19970605)54:5<461::AID-BIT6>3.0.CO;2-C
