# Supplementary material for: Glycogen phase-separation drives macromolecular rearrangement and asymmetric division in E. coli
Source: EMBO J. 2025 Nov 3;44(24):7434–76. doi: 10.1038/s44318-025-00621-y (PMC12706056; doi:10.1038/s44318-025-00621-y)
Supplement: Supplementary file 3 — Table EV3 [file 44318_2025_621_MOESM3_ESM.docx]

**Table EV3. Conditions that resulted in fluorescent protein exclusion from glycogen droplets in vitro.**

| **PEG/PEO (MW)** | **PEG/PEO**  **concentration** | **Temperature (°C)** | **FP** | **Buffer** | **FP exclusion** |
| --- | --- | --- | --- | --- | --- |
| 1.5 kDa | 60 mM | 30 | GFP | IS | Yes |
| 3 kDa | 20 mM | 25 | GFP | IS | Yes |
| 3 kDa | 20 mM | 30 | GFP | IS | Yes |
| 3 kDa | 20 mM | 37 | GFP | IS | Yes |
| 3 kDa | 20 mM | 25 | GFP | IS without MgCl_2_ | Yes |
| 3 kDa | 20 mM | 25 | mCherry | IS | Yes |
| 4 kDa | 15 mM | 25 | GFP | IS | Yes |
| 4 kDa | 15 mM | 30 | GFP | IS | Yes |
| 4 kDa | 15 mM | 37 | GFP | IS | Yes |
| 8 kDa | 3 mM | 25 | GFP | IS | Yes |
| 8 kDa | 3 mM | 30 | GFP | IS | Yes |
| 20 kDa | 750 µM | 30 | GFP | IS | Yes |
| 100 kDa | 20 µM | 25 | GFP | IS | Yes |
| 1 MDa | 10 µM | 25 | GFP | IS | Yes |

Summary of the assays performed for testing the exclusion of fluorescent proteins (FPs) by glycogen liquid condensates. FP exclusion was detected by phase contrast and fluorescence imaging. For all assays, 10 g/L of glycogen and 15 µM of the FP (GFP or mCherry) were used.
