# Supplementary material for: Glycogen phase-separation drives macromolecular rearrangement and asymmetric division in E. coli
Source: EMBO J. 2025 Nov 3;44(24):7434–76. doi: 10.1038/s44318-025-00621-y (PMC12706056; doi:10.1038/s44318-025-00621-y)
Supplement: Supplementary file 4 — Table EV4 [file 44318_2025_621_MOESM4_ESM.docx]

**Table EV4: Strain construction table**

| **Identifier** | **Genotype** | **Source or construction method** |
| --- | --- | --- |
| CJW5685 | MG1655  *rne*::*rneΔMTS-mcherry* | *rne*::*rne-mCherry-FRT-cat-FRT* from parent strain of Kti162 (Strahl et al., 2015) was moved into MG1655 by P1 transduction. Colonies were screened for chloramphenicol resistance and mCherry fluorescence. The resistance cassette was excised by transiently equipping this strain with plasmid pCP20 expressing the Flp site-specific recombinase (Cherepanov & Wackernagel, 1995). |
| CJW7083 | MG1655 *attB*::*P_proC_-msfGFP*-FRT-*kan*-FRT | pKD13 was amplified from plasmid pKD13-msfGFP (Gray et al., 2019) using primers YP32 and YP33, with YP32 encoding a transcriptional terminator sequence. *msfGFP* was also amplified from this plasmid using YP34 and YP35. Constitutive synthetic promoter *P_proC_* was amplified from CJW7006 using primers YP36 and YP37, with YP37 also encoding the ribosome binding site L3S1P13 (Chen et al., 2013), linker sequence and start codon. These pieces were assembled using Gibson assembly. The construct was then integrated into the chromosome of MG1655 at the *attB* site using lambda red recombination. |
| CJW7275 | MG1655 *attB*::*P_proC_-msfGFP* | The kanamycin resistance cassette of CJW7083 was excised by transiently equipping this strain with plasmid pCP20, expressing the Flp site specific recombinase (Cherepanov & Wackernagel, 1995). |
| CJW7325 | MG1655 *rplA*::*rplA-mCherry* *attB*::*P_proC_-msfGFP* | *attB*::PproC-msfGFP - KAN from CJW7083 was moved into CJW7324 by P1 transduction. Colonies were screened for kanamycin resistance and GFP fluorescence. The kanamycin resistance cassette was excised by transiently equipping this strain with plasmid pCP20, expressing the Flp site specific recombinase (Cherepanov & Wackernagel, 1995). |
| CJW7326 | MG1655 *attB*::*P_proC_-msfGFP* *hupA*::*hupA-mCherry* | *hupA-mCherry*-FRT-*kan*-FRT from CJW5158 (Gray et al., 2019) was moved into CJW7275 by P1 transduction. Colonies were screened for kanamycin resistance and mCherry fluorescence. The kanamycin resistance cassette was excised by transiently equipping this strain with plasmid pCP20, expressing the Flp site specific recombinase (Cherepanov & Wackernagel, 1995). |
| CJW7485 | MG1655 *rplA*::*rplA-mCherry*/pBAD-GFP(-30) | CJW7324 was transformed with plasmid pBAD-GFP(-30). |
| CJW7486 | MG1655 *rplA*::*rplA-mCherry*/pBAD-GFP(-7) | CJW7324 was transformed with plasmid pBAD-GFP(-7). |
| CJW7487 | MG1655 *rplA*::*rplA-mCherry*/pBAD-GFP(0) | CJW7324 was transformed with plasmid pBAD-GFP(0). |
| CJW7488 | MG1655 *rplA*::*rplA-mCherry*/pBAD-GFP(+7) | CJW7324 was transformed with plasmid pBAD-GFP(+7). |
| CJW7489 | MG1655 *rplA*::*rplA-mCherry*/pBAD-GFP(+11a) | CJW7324 was transformed with plasmid pBAD-GFP(+11a). |
| CJW7490 | MG1655 *rplA*::*rplA-mCherry*/pBAD-GFP(+11b) | CJW7324 was transformed with plasmid pBAD-GFP(+11b). |
| CJW7491 | MG1655 *rplA*::*rplA-mCherry*/pBAD-GFP(+15) | CJW7324 was transformed with plasmid pBAD-GFP(+15). |
| CJW7492 | MG1655 *rplA*::*rplA-mCherry*/pBAD-GFP(+25) | CJW7324 was transformed with plasmid pBAD-GFP(+25). |
| CJW7537 | MG1655 Δ*glgBXCAP*-FRT-*kan*-FRT | The glycogen operon was replaced with a kanamycin cassette using lambda red from the plasmid pSIM6 (Diner et al., 2011) using gene block gb_1. Candidate colonies we confirmed by PCR. |
| CJW7587 | MG1655 Δ*glgBXCAP* | The kanamycin resistance cassette from CJW7537 was excised by transiently equipping this strain with plasmid pCP20, expressing the Flp site specific recombinase (Cherepanov & Wackernagel, 1995). |
| CJW7588 | MG1655 *lacY(A177C) araFGH*::*spec ∆lacI ∆araE araBAD*::*dCas9 galM <PBBa-J23119-sgRNA(ftsZ)-(S. pyogenes* terminator*)-(rrnB* terminator*)> gmpA attB*::*P_proC_-msfGFP* | *attB*::*P_proC_-msfGFP*-FRT-*kan-* FRT from CJW7083 was moved into SJ_XTL229 by P1 transduction. Colonies were screened for kanamycin resistance and GFP fluorescence. The kanamycin resistance cassette was excised by transiently equipping this strain with plasmid pCP20, expressing the Flp site specific recombinase (Cherepanov & Wackernagel, 1995). |
| CJW7601 | MG1655 Tn*7*::*P_tac_-GFPmut3-CBM20* FRT-*cat-*FRT | Plasmid pNDL-1-P_tac_-GFPmut3-CBM20 FRT-CmR-FRT was transformed into MG1655. Colonies were screened for ampicillin resistance at 30°C. Integration was then carried out by incubating at 37°C and selecting for chloramphenicol resistance |
| CJW7604 | MG1655 *ΔglgBXCAP* Tn*7*::*P_tac_-GFPmut3-CBM20-*FRT*-cat-*FRT | Plasmid pNDL-1-P_tac_ -GFPmut3-CBM20 FRT-CmR-FRT was transformed into CJW7587. Colonies were screened for ampicillin resistance at 30°C. Integration was then carried out by incubating at 37°C and selecting for chloramphenicol resistance. |
| CJW7605 | MG1655 Tn*7*::*P_tac_-GFPmut3-CBM20* FRT-*cat-*FRT *hupA*::*hupA-mCherry FRT-kan-FRT* | *hupA-mCherry*-FRT-*kan-*FRT from CJW5158 (Gray et al., 2019) was moved into CJW7601 by P1 transduction. |
| CJW7606 | MG1655 Tn7:: *P_tac_ -GFPmut3-CBM20*-FRT*-cat-*FRT | CJW7601 was transformed with pEB2-mScarlet-I. |
| CJW7607 | MG1655 Δ*glgBXCAP* Tn*7*::*P_tac_-GFPmut3-CBM20* FRT-*cat* -FRT | CJW7604 was transformed with pEB2-mScarlet-I. |
| CJW7660 | MG1655 *hupA*::*hupA-mCherry* | *hupA-mCherry*-FRT-*kan-*FRT from CJW5158 (Gray et al., 2019) was moved into MG1655 by P1 transduction. Colonies were screened for kanamycin resistance and mCherry fluorescence. The kanamycin resistance cassette was excised by transiently equipping this strain with plasmid pCP20, expressing the Flp site-specific recombinase (Cherepanov & Wackernagel, 1995). |
| CJW7661 | MG1655 ∆*glgBXCAP hupA*::*hupA-mCherry* | *hupA-mCherry*-FRT-*kan-*FRT from CJW5158 (Gray et al., 2019) was moved into CJW7587 by P1 transduction. Colonies were screened for kanamycin resistance and mCherry fluorescence. The kanamycin resistance cassette was excised by transiently equipping this strain with plasmid pCP20, expressing the Flp site specific recombinase (Cherepanov & Wackernagel, 1995). |
| CJW7665 | MG1655 *hupA*::*hupA-mCherry* Tn*7*:: *P_RpsL_-mSCFP3-*FRT-*kan-*FRT | Tn*7*:: *P_RpsL_-mSCFP3-*FRT-*kan-*FRT from JP1456 was moved into CJW7660 by P1 transduction. Colonies were screened for kanamycin resistance and CFP fluorescence. |
| CJW7666 | MG1655 *hupA*::*hupA-mCherry* Tn*7*:: *P_RpsL_-mVenus-*FRT-*kan-*FRT | Tn*7*:: *P_RpsL_-mVenus-*FRT-*kan-* FRT from JP1457 was moved into CJW7660 by P1 transduction. Colonies were screened for kanamycin resistance and YFP fluorescence. |
| CJW7667 | MG1655 ∆*glgBXCAP* *hupA*::*hupA-mCherry* Tn*7*:: *P_Rps_-mSCFP3-*FRT-*kan-*FRT | Tn*7*:: *P_Rps_-mSCFP3-*FRT-*kan-*FRT from JP1456 was moved into CJW7661 by P1 transduction. Colonies were screened for kanamycin resistance and CFP fluorescence. |
| CJW7668 | MG1655 ∆*glgBXCAP* *hupA*::*hupA-mCherry* Tn*7*:: *P_Rps_-mVenus-*FRT-*kan-*FRT | Tn*7*::*P_Rps_-mVenus-*FRT-*kan-* FRT from JP1457 was moved into CJW7661 by P1 transduction. Colonies were screened for kanamycin resistance and YFP fluorescence. |
| CJW7718 | MG1655 Δ*glgBXCAP* /pEB2-mScarlet-I | CJW7587 was transformed with plasmid pEB2-mScarlet-I from AddGene #104007 (Balleza et al., 2018). |
| CJW7872 | MG1655 *ΔminD minE::sfgfp-minD minE::frt kanR frt* | *ΔminD minE::sfgfp-minD minE::frt kanR frt* was moved into MG1655 by P1 transduction from FW1537 (Wu et al., 2015). |
| CJW7877 | MG1655 ∆*glgBXCAP rne*::*rneΔMTS-mCherry* | Δ*glgBXCAP*-FRT-*kan*-FRT was moved into CJW5685 by P1 transduction from CJW7537. |
| CJW7878 | MG1655 ∆*glgBXCAP rplA*::*rplA-mCherry* *attB*::*P_proC_-msfGFP* | Δ*glgBXCAP*-FRT-*kan*-FRT was moved into CJW7325 by P1 transduction from CJW7537. |

**References**

Balleza, E., Kim, J. M., & Cluzel, P. (2018). Systematic characterization of maturation time of fluorescent proteins in living cells. *Nature Methods*, *15*(1), 47–51. https://doi.org/10.1038/nmeth.4509

Chen, Y. J., Liu, P., Nielsen, A. A. K., Brophy, J. A. N., Clancy, K., Peterson, T., & Voigt, C. A. (2013). Characterization of 582 natural and synthetic terminators and quantification of their design constraints. *Nature Methods 2013 10:7*, *10*(7), 659–664. https://doi.org/10.1038/nmeth.2515

Cherepanov, P. P., & Wackernagel, W. (1995). Gene disruption in Escherichia coli: TcR and KmR cassettes with the option of Flp-catalyzed excision of the antibiotic-resistance determinant. *Gene*, *158*(1), 9–14. https://doi.org/10.1016/0378-1119(95)00193-A

Diner, E. J., Garza-Sánchez, F., & Hayes, C. S. (2011). Genome engineering using targeted oligonucleotide libraries and functional selection. *Methods in Molecular Biology (Clifton, N.J.)*, *765*(1), 71–82. https://doi.org/10.1007/978-1-61779-197-0_5

Gray, W. T., Govers, S. K., Xiang, Y., Parry, B. R., Campos, M., Kim, S., & Jacobs-Wagner, C. (2019). Nucleoid size scaling and intracellular organization of translation across bacteria. *Cell*, *177*(6), 1632-1648.e20. https://doi.org/10.1016/j.cell.2019.05.017

Strahl, H., Turlan, C., Khalid, S., Bond, P. J., Kebalo, J.-M., Peyron, P., Poljak, L., Bouvier, M., Hamoen, L., Luisi, B. F., & Carpousis, A. J. (2015). Membrane recognition and dynamics of the RNA degradosome. *PLoS Genetics*, *11*(2), e1004961. https://doi.org/10.1371/journal.pgen.1004961

Wu, F., van Schie, B. G. C., Keymer, J. E., & Dekker, C. (2015). Symmetry and scale orient Min protein patterns in shaped bacterial sculptures. *Nature Nanotechnology*, *10*(8), 719–726. https://doi.org/10.1038/nnano.2015.126
