# Supplementary material for: Glycogen phase-separation drives macromolecular rearrangement and asymmetric division in E. coli
Source: EMBO J. 2025 Nov 3;44(24):7434–76. doi: 10.1038/s44318-025-00621-y (PMC12706056; doi:10.1038/s44318-025-00621-y)
Supplement: Supplementary file 5 — Table EV5 [file 44318_2025_621_MOESM5_ESM.docx]

**Table EV5. gBlocks used in this study.**

| **Identifier** | **Sequence (5’ to 3’)** |
| --- | --- |
| pNDL-1-Ptac-GFPmut3-CBM20 FRT-CmR-FRT | CACCGAATTCCCCGGGGGGGACAAGTTTGTACAAAAAAGCAGGCTTACGACATCATAACGGTTCTGGCAAATATTCTGAAATGAGCTGTTGACAATTAATCATCGGCTCGTATAATGTGTGGAATTGTGAGCGGATAACAATTTCACACAGGAAACAGAATATGAGTAAAGGAGAAGAACTTTTCACTGGAGTTGTCCCAATTCTTGTTGAATTAGATGGTGATGTTAATGGGCACAAATTTTCTGTCAGTGGAGAGGGTGAAGGTGATGCAACATACGGAAAACTTACCCTTAAATTTATTTGCACTACTGGAAAACTACCTGTTCCATGGCCAACACTTGTCACTACTTTCGGTTATGGTGTTCAATGCTTTGCGAGATACCCAGATCATATGAAACAGCATGACTTTTTCAAGAGTGCCATGCCCGAAGGTTATGTACAGGAAAGAACTATATTTTTCAAAGATGACGGGAACTACAAGACACGTGCTGAAGTCAAGTTTGAAGGTGATACCCTTGTTAATAGAATCGAGTTAAAAGGTATTGATTTTAAAGAAGATGGAAACATTCTTGGACACAAATTGGAATACAACTATAACTCACACAATGTATACATCATGGCAGACAAACAAAAGAATGGAATCAAAGTTAACTTCAAAATTAGACACAACATTGAAGATGGAAGCGTTCAACTAGCAGACCATTATCAACAAAATACTCCAATTGGCGATGGCCCTGTCCTTTTACCAGACAACCATTACCTGTCCACACAATCTAAGCTTTCGAAAGATCCCAACGAAAAGAGAGACCACATGGTCCTTCTTGAGTTTGTAACAGCTGCTGGGATTACACATGGCATGGATGAACTATACAAAAGCGATTTCATGCCTGCCGGTTCTCAACAGGTTAGCGTTCGTTTTCAGGTACACTACGTCACTTCCACCGACGTTCAGTTTATTGCAGTGACCGGCGATCACGAGTGCCTGGGCCGCTGGAACACGTACATCCCTCTGCACTACAACAAAGACGGTTTCTGGTCCCACAGCATCTTTCTGCCGGCGGATACCGTTGTGGAGTGGAAATTTGTTCTGGTTGAAAACGGTGGCGTTACGCGTTGGGAGGAATGTTCCAACCGTTTCCTGGAAACCGGTCACGAAGATAAGGTAGTTCACGCGTGGTGGGGTATCCATTAAATGTCCAGACCTGCAGGCATGCAAGCTCTAGTTGGATTCTCACCAATAAAAAACGCCCGGCGGCAACCGAGCGTTCTGAACAAATCCAGATGGAGTTCTGAGGTCATTACTGGATCTATCAACAGGAGTCCAAGCGAGCGAATTCTGCAGGTGTTAATTCAGGAGCATTGTTATCAGACCAAATATGTGTAGGCTGGAGCTGCTTCGAAGTTCCTATACTTTCTAGAGAATAGGAACTTCGGAATAGGAACTTCTTATCAAAAAGGATCTTCACCTAGATCCTTTTAAATTAAAAATGAAGTTTTAAATCAATCTAAAGTATATATGAGTAAACTTGGTCTGACAGCTCGAGGCGCTCGATATCAAATTACGCCCCGCCCTGCCACTCATCGCAGTACTGTTGTAATTCATTAAGCATTCTGCCGACATGGAAGCCATCACAAACGGCATGATGAACCTGAATCGCCAGCGGCATCAGCACCTTGTCGCCTTGCGTATAATATTTGCCCATGGTGAAAACGGGGGCGAAGAAGTTGTCCATATTGGCCACGTTTAAATCAAAACTGGTGAAACTCACCCAGGGATTGGCTGAGACGAAAAACATATTCTCAATAAACCCTTTAGGGAAATAGGCCAGGTTTTCACCGTAACACGCCACATCTTGCGAATATATGTGTAGAAACTGCCGGAAATCGTCGTGGTATTCACTCCAGAGCGATGAAAACGTTTCAGTTTGCTCATGGAAAACGGTGTAACAAGGGTGAACACTATCCCATATCACCAGCTCACCGTCTTTCATTGCCATACGAAATTCCGGATGAGCATTCATCAGGCGGGCAAGAATGTGAATAAAGGCCGGATAAAACTTGTGCTTATTTTTCTTTACGGTCTTTAAAAAGGCCGTAATATCCAGCTGAACGGTCTGGTTATAGGTACATTGAGCAACTGACTGAAATGCCTCAAAATGTTCTTTACGATGCCATTGGGATATATCAACGGTGGTATATCCAGTGATTTTTTTCTCCATTTTAGCTTCCTTAGCTCCTGAAAATCTCGATAACTCAAAAAATACGCCCGGTAGTGATCTTATTTCATTATGGTGAAAGTTGGAACCTCTTACGTGCCCGATCAACTCGAGTGCCACCTGACGTCTAAGAAACCATTATTATCATGACATTAACCTATAAAAATAGGCGTATCACGAGGCAGAATTTCAGATAAAAAAAATCCTTAGCTTTCGCTAAGGATGATTTCTGGAAGCGCTCTGAAGTTCCTATACTTTCTAGAGAATAGGAACTTCGTACCCAGCTTTCTTGTACAAAGTGGTCCCCAAGCTTCTGCAGAGCT |
| gb_1 | TCAATAATACCGGCAAGTCCGACACCCAGCATGGCAATAACCACCGCCAAAAATTGCGCCAGTATGGGGATGCCGAAAAAAGTCATTACCAGCGAGGTCAAAATCCATTTTCTGTTTTGCATTATTCTTTCCATTCTTTTTGAATGGTGAAATTATACTCCCCGAGTCCCCTTGCCCCTTCTGGACACTTTTCCGAAATGATGGCGGAAAAAAACGGGACCCTTTGGCCCCGTTCTATTTATTGGTGAACGTGTAGGCTGGAGCTGCTTCGAAGTTCCTATACTTTCTAGAGAATAGGAACTTCGGAATAGGAACTTCAAGATCCCCTTATTAGAAGAACTCGTCAAGAAGGCGATAGAAGGCGATGCGCTGCGAATCGGGAGCGGCGATACCGTAAAGCACGAGGAAGCGGTCAGCCCATTCGCCGCCAAGCTCTTCAGCAATATCACGGGTAGCCAACGCTATGTCCTGATAGCGGTCCGCCACACCCAGCCGGCCACAGTCGATGAATCCAGAAAAGCGGCCATTTTCCACCATGATATTCGGCAAGCAGGCATCGCCATGGGTCACGACGAGATCCTCGCCGTCGGGCATGCGCGCCTTGAGCCTGGCGAACAGTTCGGCTGGCGCGAGCCCCTGATGCTCTTCGTCCAGATCATCCTGATCGACAAGACCGGCTTCCATCCGAGTACGTGCTCGCTCGATGCGATGTTTCGCTTGGTGGTCGAATGGGCAGGTAGCCGGATCAAGCGTATGCAGCCGCCGCATTGCATCAGCCATGATGGATACTTTCTCGGCAGGAGCAAGGTGAGATGACAGGAGATCCTGCCCCGGCACTTCGCCCAATAGCAGCCAGTCCCTTCCCGCTTCAGTGACAACGTCGAGCACAGCTGCGCAAGGAACGCCCGTCGTGGCCAGCCACGATAGCCGCGCTGCCTCGTCCTGCAGTTCATTCAGGGCACCGGACAGGTCGGTCTTGACAAAAAGAACCGGGCGCCCCTGCGCTGACAGCCGGAACACGGCGGCATCAGAGCAGCCGATTGTCTGTTGTGCCCAGTCATAGCCGAATAGCCTCTCCACCCAAGCGGCCGGAGAACCTGCGTGCAATCCATCTTGTTCAATCATGCGAAACGATCCTCATCCTGTCTCTTGATCAGATCTTGATCCCCTGCGCCATCAGATCCTTGGCGGCAAGAAAGCCATCCAGTTTACTTTGCAGGGCTTCCCAACCTTACCAGAGGGCGCCCCAGCTGGCAATTCCGGTTCGCTTGCTGTCCATAAAACCGCCCAGTCTAGCTATCGCCATGTAAGCCCACTGCAAGCTACCTGCTTTCTCTTTGCGCTTGCGTTTTCCCTTGTCCAGATAGCCCAGTAGCTGACATTCATCCGGGGTCAGCACCGTTTCTGCGGACTGGCTTTCTACGTGTTCCGCTTCCTTTAGCAGCCCTTGCGCCCTGAGTGCTTGCGGCAGCGTGAGCTTCAAAAGCGCTCTGAAGTTCCTATACTTTCTAGAGAATAGGAACTTCGGCTTGTCTTCCTGTTTTATTGTGTCACCCCATCCAATTGGATTTTTGACTTCTGGTTGTGACATTTTTTTGACCTGAACGGCGCAGCACTCTGTGCATCCTCTCTGCGTCGTCCTCACTTCAGGTAAGGCTGTGAATACTCATGTATTCAGCCACCCTTAAAGAATAGCCAATGCTCTATTTAACTCCCGGTAAATCATGAAACATCTGCGCTTACTCCTGTATTACGCACTAACAGGGGCGGCATCGCGCCCCAGATTTAATGAATAAAGATTACGCCAGTT |
| gb_2 | CACCGAATTCCCCGGGGGGGACAAGTTTGTACAAAAAAGCAGGCTTACGACATCATAACGGTTCTGGCAAATATTCTGAAATGAGCTGTTGACAATTAATCATCGGCTCGTATAATGTGTGGAATTGTGAGCGGATAACAATTTCACACAGGAAACAGAATATGAGTAAAGGAGAAGAACTTTTCACTGGAGTTGTCCCAATTCTTGTTGAATTAGATGGTGATGTTAATGGGCACAAATTTTCTGTCAGTGGAGAGGGTGAAGGTGATGCAACATACGGAAAACTTACCCTTAAATTTATTTGCACTACTGGAAAACTACCTGTTCCATGGCCAACACTTGTCACTACTTTCGGTTATGGTGTTCAATGCTTTGCGAGATACCCAGATCATATGAAACAGCATGACTTTTTCAAGAGTGCCATGCCCGAAGGTTATGTACAGGAAAGAACTATATTTTTCAAAGATGACGGGAACTACAAGACACGTGCTGAAGTCAAGTTTGAAGGTGATACCCTTGTTAATAGAATCGAGTTAAAAGGTATTGATTTTAAAGAAGATGGAAACATTCTTGGACACAAATTGGAATACAACTATAACTCACACAATGTATACATCATGGCAGACAAACAAAAGAATGGAATCAAAGTTAACTTCAAAATTAGACACAACATTGAAGATGGAAGCGTTCAACTAGCAGACCATTATCAACAAAATACTCCAATTGGCGATGGCCCTGTCCTTTTACCAGACAACCATTACCTGTCCACACAATCTAAGCTTTCGAAAGATCCCAACGAAAAGAGAGACCACATGGTCCTTCTTGAGTTTGTAACAGCTGCTGGGATTACACATGGCATGGATGAACTATACAAAAGCGATTTCATGCCTGCCGGTTCTCAACAGGTTAGCGTTCGTTTTCAGGTACACTACGTCACTTCCACCGACGTTCAGTTTATTGCAGTGACCGGCGATCACGAGTGCCTGGGCCGCTGGAACACGTACATCCCTCTGCACTACAACAAAGACGGTTTCTGGTCCCACAGCATCTTTCTGCCGGCGGATACCGTTGTGGAGTGGAAATTTGTTCTGGTTGAAAACGGTGGCGTTACGCGTTGGGAGGAATGTTCCAACCGTTTCCTGGAAACCGGTCACGAAGATAAGGTAGTTCACGCGTGGTGGGGTATCCATTAAATGTCCAGACCTGCAGGCATGCAAGCTCTAGTTGGATTCTCACCAATAAAAAACGCCCGGCGGCAACCGAGCGTTCTGAACAAATCCAGATGGAGTTCTGAGGTCATTACTGGATCTATCAACAGGAGTCCAAGCGAGCGAATTCTGCAGGTGTTAATTCAGGAGCATTGTTATCAGACCAAATATGTGTAGGCTGGAGCTGCTTCGAAGTTCCTATACTTTCTAGAGAATAGGAACTTCGGAATAGGAACTTCTTATCAAAAAGGATCTTCACCTAGATCCTTTTAAATTAAAAATGAAGTTTTAAATCAATCTAAAGTATATATGAGTAAACTTGGTCTGACAGCTCGAGGCGCTCGATATCAAATTACGCCCCGCCCTGCCACTCATCGCAGTACTGTTGTAATTCATTAAGCATTCTGCCGACATGGAAGCCATCACAAACGGCATGATGAACCTGAATCGCCAGCGGCATCAGCACCTTGTCGCCTTGCGTATAATATTTGCCCATGGTGAAAACGGGGGCGAAGAAGTTGTCCATATTGGCCACGTTTAAATCAAAACTGGTGAAACTCACCCAGGGATTGGCTGAGACGAAAAACATATTCTCAATAAACCCTTTAGGGAAATAGGCCAGGTTTTCACCGTAACACGCCACATCTTGCGAATATATGTGTAGAAACTGCCGGAAATCGTCGTGGTATTCACTCCAGAGCGATGAAAACGTTTCAGTTTGCTCATGGAAAACGGTGTAACAAGGGTGAACACTATCCCATATCACCAGCTCACCGTCTTTCATTGCCATACGAAATTCCGGATGAGCATTCATCAGGCGGGCAAGAATGTGAATAAAGGCCGGATAAAACTTGTGCTTATTTTTCTTTACGGTCTTTAAAAAGGCCGTAATATCCAGCTGAACGGTCTGGTTATAGGTACATTGAGCAACTGACTGAAATGCCTCAAAATGTTCTTTACGATGCCATTGGGATATATCAACGGTGGTATATCCAGTGATTTTTTTCTCCATTTTAGCTTCCTTAGCTCCTGAAAATCTCGATAACTCAAAAAATACGCCCGGTAGTGATCTTATTTCATTATGGTGAAAGTTGGAACCTCTTACGTGCCCGATCAACTCGAGTGCCACCTGACGTCTAAGAAACCATTATTATCATGACATTAACCTATAAAAATAGGCGTATCACGAGGCAGAATTTCAGATAAAAAAAATCCTTAGCTTTCGCTAAGGATGATTTCTGGAAGCGCTCTGAAGTTCCTATACTTTCTAGAGAATAGGAACTTCGTACCCAGCTTTCTTGTACAAAGTGGTCCCCAAGCTTCTGCAGAGCT |
