# Supplementary material for: Glycogen phase-separation drives macromolecular rearrangement and asymmetric division in E. coli
Source: EMBO J. 2025 Nov 3;44(24):7434–76. doi: 10.1038/s44318-025-00621-y (PMC12706056; doi:10.1038/s44318-025-00621-y)
Supplement: Supplementary file 7 — Movie EV1 [file 44318_2025_621_MOESM7_ESM.zip › Movie_EV1/MovieEV1_MovieLegend.docx]

**Video EV1: Timelapse sequence showing that nucleoids and ribosomes remain closer to the new pole upon division during transition phase.**

Montage video showing phase and fluorescence overlays of a representative cell expressing RplA-msGFP and HupA-mCherry (CJW5159) growing and dividing in transition phase. Cells from a culture in late exponential phase (OD = 0.5) were washed in transition phase medium, spotted on an agarose pad containing transition phase medium (OD = 2.63), and imaged every 2 min. Time stamp shows h:min:s.
