# Supplementary material for: Glycogen phase-separation drives macromolecular rearrangement and asymmetric division in E. coli
Source: EMBO J. 2025 Nov 3;44(24):7434–76. doi: 10.1038/s44318-025-00621-y (PMC12706056; doi:10.1038/s44318-025-00621-y)
Supplement: Supplementary file 8 — Movie EV2 [file 44318_2025_621_MOESM8_ESM.zip › Movie_EV2/MovieEV2_MovieLegend.docx]

**Video EV2: Timelapse of the preferential glycogen accumulation at the old cell pole through asymmetric inheritance**

Timelapse of representative mother cell lineage of CJW7605 showing the overlay between the phase contrast, HupA-mCherry and glycogen sensor signals. Scale bar: 2 µm. Time stamp shows h:min.
