# Supplementary material for: Glycogen phase-separation drives macromolecular rearrangement and asymmetric division in E. coli
Source: EMBO J. 2025 Nov 3;44(24):7434–76. doi: 10.1038/s44318-025-00621-y (PMC12706056; doi:10.1038/s44318-025-00621-y)
Supplement: Supplementary file 9 — Movie EV3 [file 44318_2025_621_MOESM9_ESM.zip › Movie_EV3/MovieEV3_MovieLegend.docx]

**Video EV3: Timelapse of the MinD-GFP oscillations.**

Video of CJW7872 cells in transition phase. *Left*, Overlay between phase contrast and DAPI at time zero. *Right*, Timelapse of the MinD-GFP fluorescence channel (frame rate = 5 seconds). Scale bar: 1 µm. Time stamp shows min:s.
