# Supplementary material for: Glycogen phase-separation drives macromolecular rearrangement and asymmetric division in E. coli
Source: EMBO J. 2025 Nov 3;44(24):7434–76. doi: 10.1038/s44318-025-00621-y (PMC12706056; doi:10.1038/s44318-025-00621-y)
Supplement: Supplementary file 10 — Movie EV4 [file 44318_2025_621_MOESM10_ESM.zip › Movie_EV4/MovieEV4_MovieLegend.docx]

**Video EV4: Timelapse sequence showing glycogen condensate fusions in vitro.**

Timelapse sequence of glycogen droplets labeled with ConA-FITC as they fuse. The sample was made with 10 g/L of glycogen and 20 mM of 3 kDa PEG in IS buffer. Phase contrast (left) and FITC (right) images were acquired every 10 s at 25°C. Time stamp shows h:min:s.
