# Supplementary material for: Glycogen phase-separation drives macromolecular rearrangement and asymmetric division in E. coli
Source: EMBO J. 2025 Nov 3;44(24):7434–76. doi: 10.1038/s44318-025-00621-y (PMC12706056; doi:10.1038/s44318-025-00621-y)
Supplement: Supplementary file 11 — Movie EV5 [file 44318_2025_621_MOESM11_ESM.zip › Movie_EV5/MovieEV5_MovieLegend.docx]

**Video EV5: Timelapse sequence of glycogen droplets.**

Timelapse sequence showing the phase-separated glycogen droplets undergoing fusion events and exhibiting surface wetting. The sample was made with 10 g/L of glycogen and 750 µM of 20kDa PEG in the IS buffer. Phase-contrast images were acquired every 30 s at 25°C. Time stamp shows min:s.
