# Supplementary material for: Glycogen phase-separation drives macromolecular rearrangement and asymmetric division in E. coli
Source: EMBO J. 2025 Nov 3;44(24):7434–76. doi: 10.1038/s44318-025-00621-y (PMC12706056; doi:10.1038/s44318-025-00621-y)
Supplement: Supplementary file 12 — Movie EV6 [file 44318_2025_621_MOESM12_ESM.zip › Movie_EV6/MovieEV6_MovieLegend.docx]

**Video EV6: Timelapse sequence of “collapsed” glycogen aggregates.**

Timelapse sequence showing the “collapsed” phase-separated glycogen undergoing collisions that do not result in fusion events. The sample was made with 10 g/L of glycogen and 30 mM of 3kDa PEG in the IS buffer. Phase-contrast images were acquired every 10 s at 25°C. Time stamp shows h:min:s.
