# Supplementary material for: Glycogen phase-separation drives macromolecular rearrangement and asymmetric division in E. coli
Source: EMBO J. 2025 Nov 3;44(24):7434–76. doi: 10.1038/s44318-025-00621-y (PMC12706056; doi:10.1038/s44318-025-00621-y)
Supplement: Supplementary file 13 — Movie EV7 [file 44318_2025_621_MOESM13_ESM.zip › Movie_EV7/MovieEV7_MovieLegend.docx]

**Video EV7: Z-stack sequence of glycogen condensates excluding GFP.** Z-stacks of both phase contrast and GFP of glycogen droplets floating in the sample. Each frame corresponds to a step of 0.125 µm, starting from a point 1.75 µm above the glass surface (z = 0 nm). The sample was prepared with glycogen (10 g/L) in IS buffer mixed with GFP (15 µM) and 100 kDa PEG (200 µM). The imaging was performed at 30°C.
