# Supplementary material for: Glycogen phase-separation drives macromolecular rearrangement and asymmetric division in E. coli
Source: EMBO J. 2025 Nov 3;44(24):7434–76. doi: 10.1038/s44318-025-00621-y (PMC12706056; doi:10.1038/s44318-025-00621-y)
Supplement: Supplementary file 32 — Expanded View Figures [file 44318_2025_621_MOESM32_ESM.pdf]

Expanded View Figures

Figure EV1. Localization of various cytoplasmic probes in cells in transition phase.

(**A**) Representative fluorescence images of DAPI-stained *E. coli* cells expressing different ribosomal protein fusions in transition phase: RplA-GFP (CJW4677), RplA-msfGFP (CJW7020), and RpsB-msfGFP (CJW7021). Arrowheads indicate cell areas of ribosome signal depletion. Scale bar: 2 µm. (**B**) Representative fluorescence image of DAPI-stained *E. coli* cells (CJW7006) expressing mScarlet-I in transition phase. Arrowheads indicate cell areas of mScarlet-I depletion. Scale bar: 2 µm. (**C**) Representative fluorescence images of DAPI-stained *E. coli* expressing RplA-mCherry as well as free fluorescent GFP variants with the following net surface charges (strain name): -30 (CJW7485), -7 (CJW7486), 0 (CJW7487), +7 (CJW7488), +11a (CJW7489), +11b (CJW7490), +15 (CJW7491), and +25 (CJW7492). For GFP with a net surface charge +11, 'a' and 'b' refer to variations in the distribution of the charge on the protein surface (Schavemaker et al, 2017). Cells were grown in M9gluCAAT and supplemented with 0.4% arabinose to induce GFP expression. Arrowheads indicate cell areas depleted of the GFP variant. Scale bar: 2 µm. (**D**) Fluorescence images of representative DAPI- and RNASelect-labeled *E. coli* cells (CJW7324) in exponential (OD ~ 0.30) and transition (OD ~ 1.72) phase. Also shown are the corresponding fluorescence signal profiles for the indicated (*) cells. Arrowheads indicate areas depleted of RNASelect signal. Scale bar: 2 µm. Source data are available online for this figure.

© The Author(s)

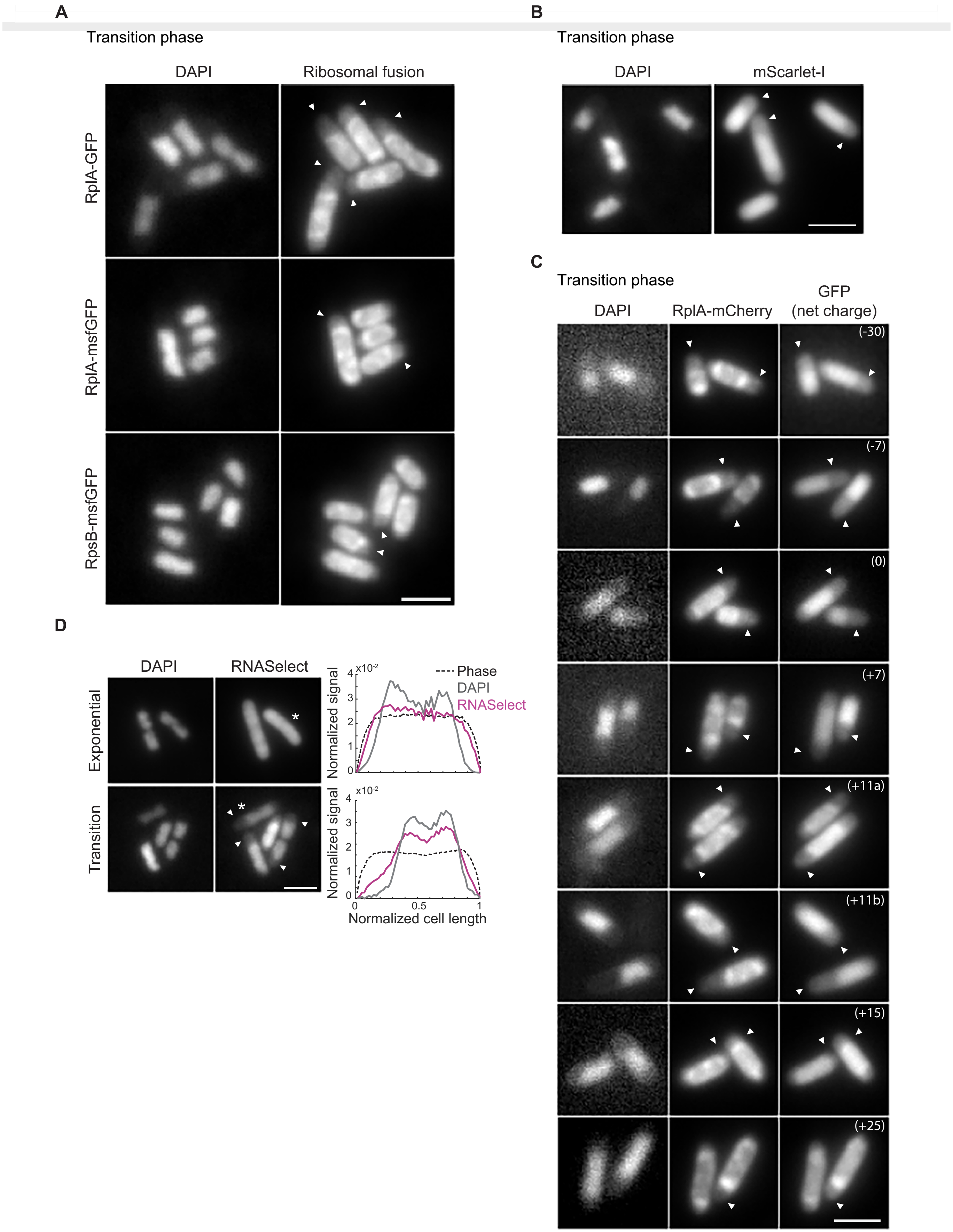
A
Transition phase
DAPI
Ribosomal fusion
RplA-GFP
RplA-msfGFP
RpsB-msfGFP
B
Transition phase
DAPI
mScarlet-I
C
Transition phase
DAPI
RplA-mCherry
GFP (net charge)
(-30)
(-7)
(0)
(+7)
(+11a)
(+11b)
(+15)
(+25)
D
DAPI
RNASelect
Exponential
Transition
Phase
DAPI
RNASelect
Normalized signal
x10-2
Normalized cell length

 © The Author(s)

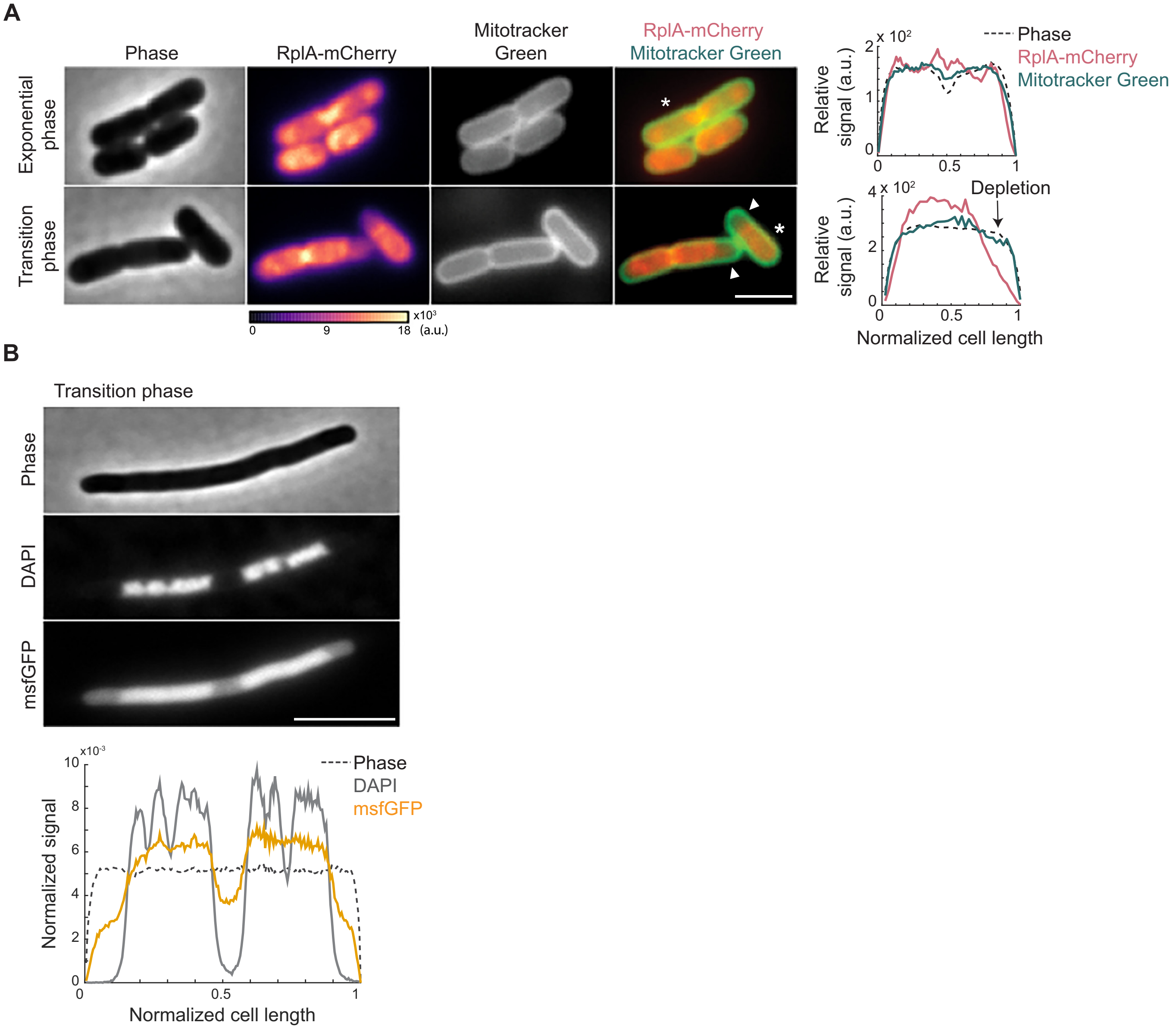

Figure EV2. Assessment of the potential effects of membrane retraction and cell division on the localization of cytoplasmic probes in transition-phase cells.

(**A**) Microscopy images of MitoTracker Green-labeled cells (CJW7324) expressing RplA-mCherry. The samples were obtained from cultures in either exponential or transition phase. Fluorescence intensities are indicated in arbitrary units (a.u.). Signal intensity profiles are provided for the cells indicated by asterisks. White arrowheads show the depletion of RplA-mCherry signal at a cell pole. (**B**) Representative microscopy images of DAPI-stained FtsZ-depleted cells (CJW7588) expressing cytoplasmic msfGFP in transition phase in M9gluCAAT supplemented with 0.4% arabinose to induce the CRISPRi system, thereby blocking the expression of *ftsZ*. The corresponding signal intensity profile is shown below. Scale bar: 5 µm. Source data are available online for this figure.

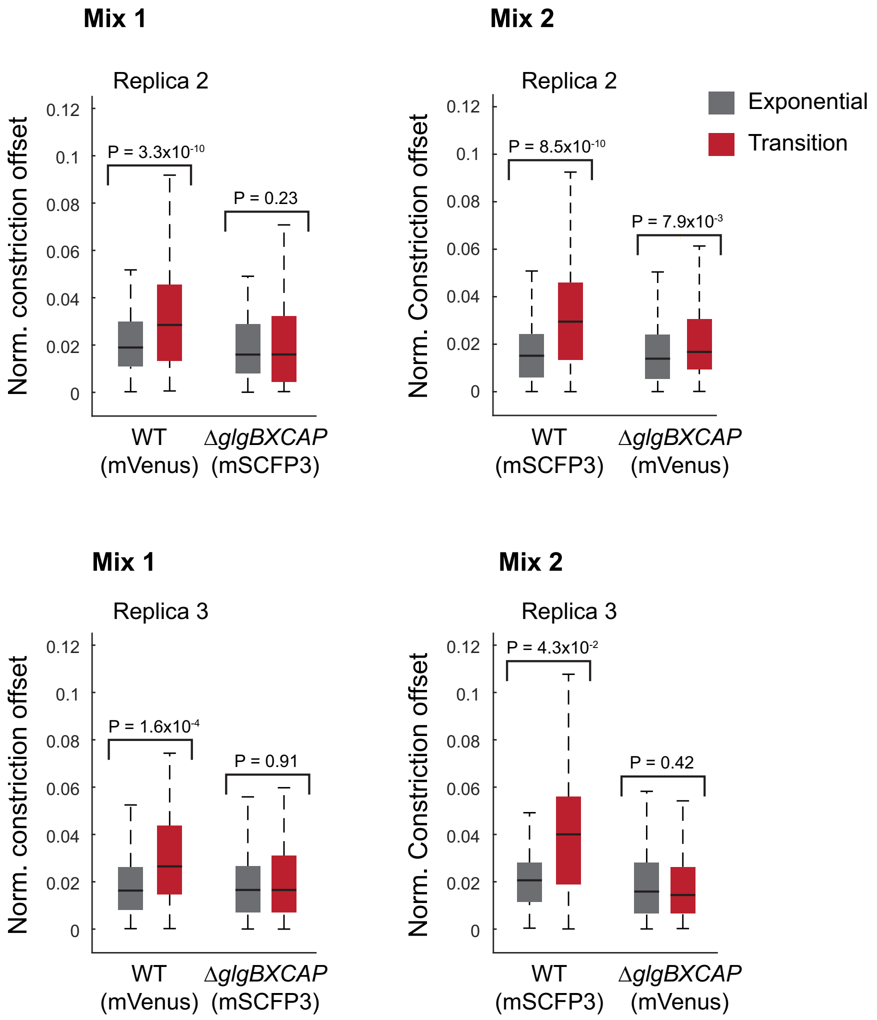

Figure EV3. Normalized constriction offset measurements for the WT and Δ*glgBXCAP* cells from co-culture experiments.

Boxplots of the normalized constriction offset in exponential and transition phases for the biological replicates of the co-culture experiments shown in Fig. 3C. The horizontal lines in the boxes correspond to the medians, with the bottom and top of the boxes showing the 25th and 75th percentiles, respectively. The endpoints of the whiskers mark the minimum and maximum values within a range that excludes the outliers. Outlier values are defined as those more than 1.5 times the interquartile range away from the bottom or top of the boxes. The indicated P values were obtained using a two-sided Wilcoxon rank sum test. Source data are available online for this figure.

© The Author(s)

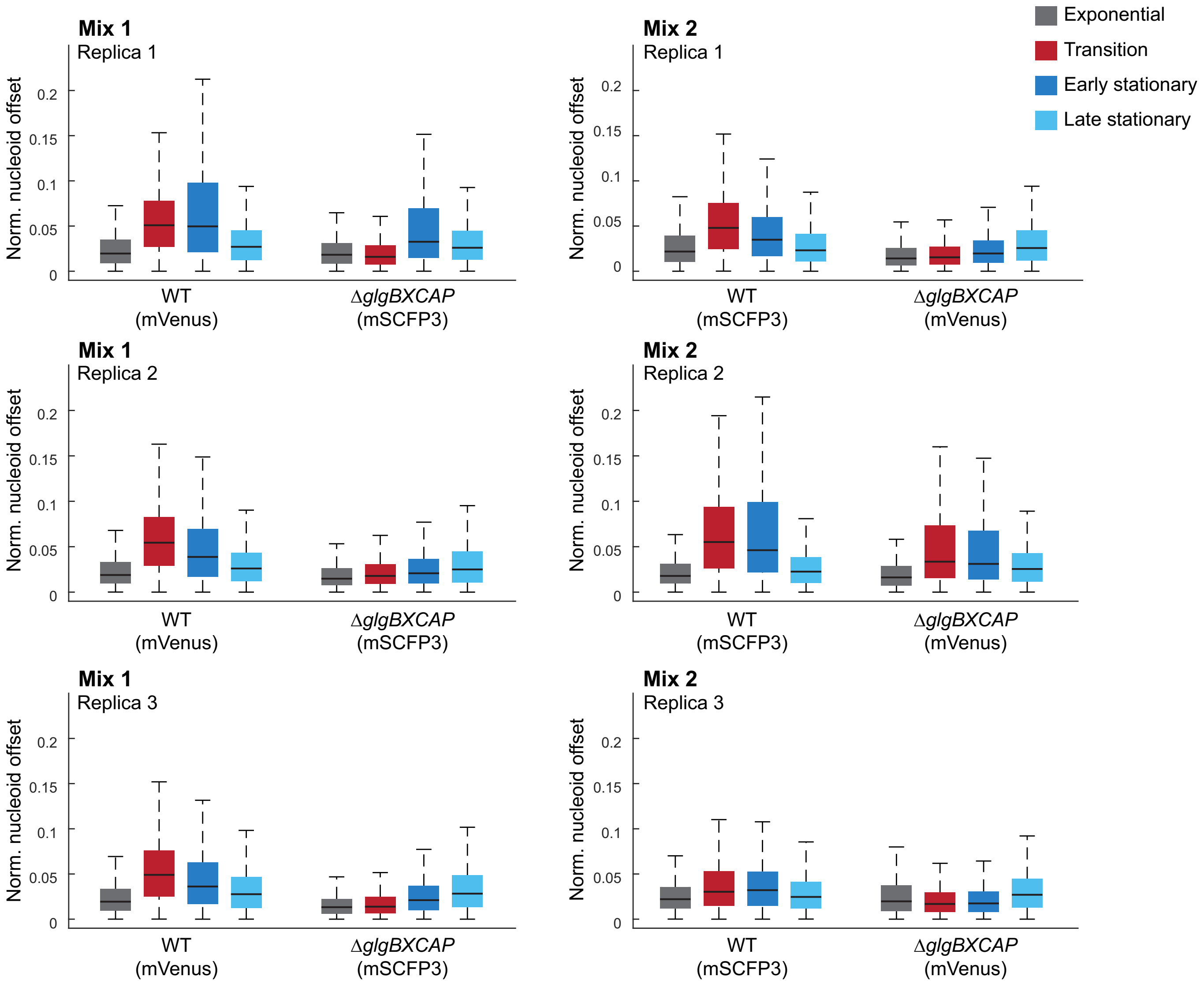

Figure EV4. Normalized nucleoid offset measurements for the WT and Δ*glgBXCAP* cells from the co-culture experiments across different growth phases.

Boxplots of the normalized nucleoid offset in exponential, transition, early stationary (24 h), and late stationary phase (72 h) for all the biological replicates of the co-culture experiments. Exponential and transition phase datapoints for Replica 1 of both mixes are shown in Fig. 3D. The horizontal lines in the boxes correspond to the medians, with the bottom and top of the boxes showing the 25th and 75th percentiles, respectively. The endpoints of the whiskers mark the minimum and maximum values within a range that excludes the outliers. Outlier values are defined as those more than 1.5 times the interquartile range away from the bottom or top of the boxes. The indicated *P* values were obtained using a two-sided Wilcoxon rank-sum test. Source data are available online for this figure.

© The Author(s)

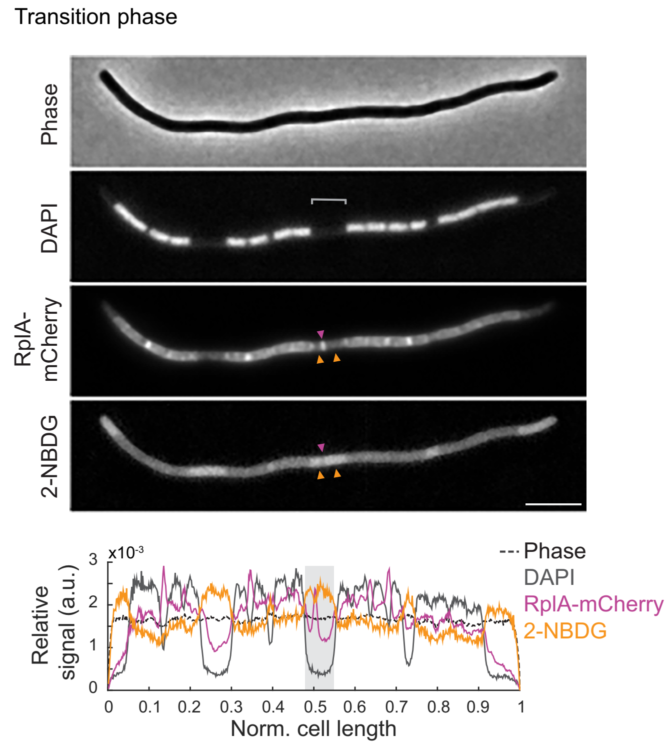

Figure EV5. Representative image of 2-NBDG incorporation in a filamentous polynucleoid cell in transition phase.

Microscopy images of a cephalexin-treated cell (CJW7324) expressing RplA-mCherry in transition phase (OD ~ 2.0). Yellow arrowheads indicate accumulations of 2-NBDG that sandwich an accumulation of RplA-mCherry signal. Below is the cell signal intensity profile, with the gray shade highlighting the region of interest. Scale bar: 5 µm. Source data are available online for this figure.

 © The Author(s)

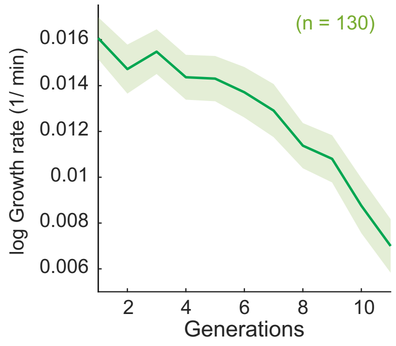

Figure EV6. Growth rate measurements in the microfluidic device.

Plot of the calculated log growth rate as a function of cell generations for the strain CJW7605 ($n = 130$ lineages). The solid line and shaded region correspond to the average and the 95% confidence interval, respectively. Source data are available online for this figure.

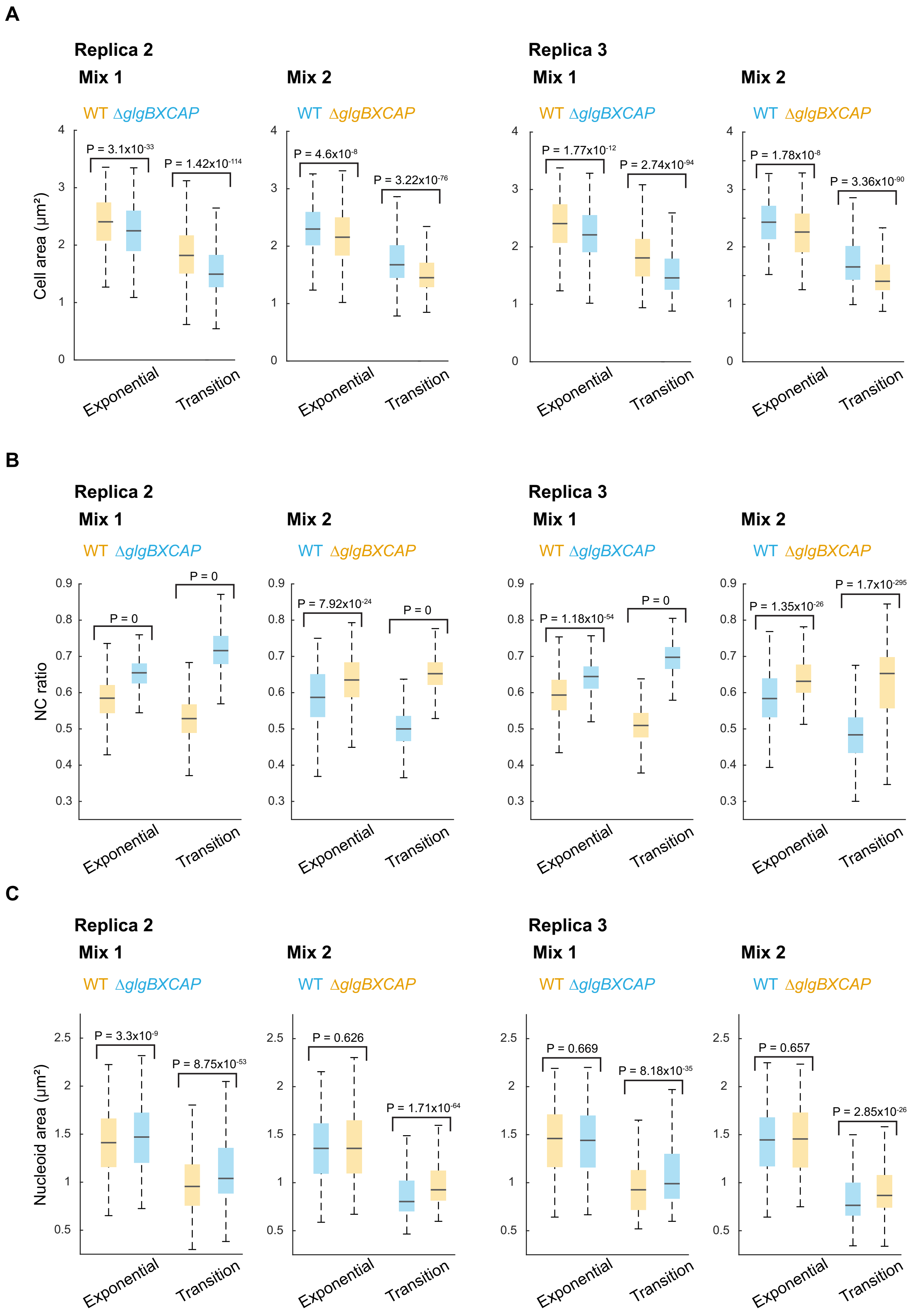
A
Replica 2
Mix 1
WT ΔglgBXCAP
P = 3.1x10-33
P = 1.42x10-114
Cell area (µm²)
Exponential
Transition
Mix 2
WT ΔglgBXCAP
P = 4.6x10-8
P = 3.22x10-76
Replica 3
Mix 1
WT ΔglgBXCAP
P = 1.77x10-12
P = 2.74x10-94
Mix 2
WT ΔglgBXCAP
P = 1.78x10-8
P = 3.36x10-90
B
Replica 2
Mix 1
WT ΔglgBXCAP
P = 0
P = 0
NC ratio
Mix 2
WT ΔglgBXCAP
P = 7.92x10-24
P = 0
Replica 3
Mix 1
WT ΔglgBXCAP
P = 1.18x10-54
P = 0
Mix 2
WT ΔglgBXCAP
P = 1.35x10-26
P = 1.7x10-295
C
Replica 2
Mix 1
WT ΔglgBXCAP
P = 3.3x10-9
P = 8.75x10-53
Nucleoid area (µm²)
Mix 2
WT ΔglgBXCAP
P = 0.626
P = 1.71x10-64
Replica 3
Mix 1
WT ΔglgBXCAP
P = 0.669
P = 8.18x10-35
Mix 2
WT ΔglgBXCAP
P = 0.657
P = 2.85x10-26

 © The Author(s)

Figure EV7. Cell area, NC ratio and nucleoid area measurements for the WT and Δ*glgBXCAP* cells from co-culture experiments.

(**A**) Boxplots of the cell area of exponential and transition-phase cells for the biological replicates of the co-culture experiments shown in Fig. 5. The horizontal lines in the boxes correspond to the medians, with the bottom and top of the boxes showing the 25th and 75th percentiles, respectively. The endpoints of the whiskers mark the minimum and maximum values within a range that excludes the outliers. Outlier values are defined as those more than 1.5 times the interquartile range away from the bottom or top of the boxes. The indicated *P* values were obtained using a two-sided Wilcoxon rank-sum test. (**B**) Same as (**A**) but for the NC ratio. (**C**) Same as (**A**) but for the total nucleoid area. Source data are available online for this figure.

© The Author(s)

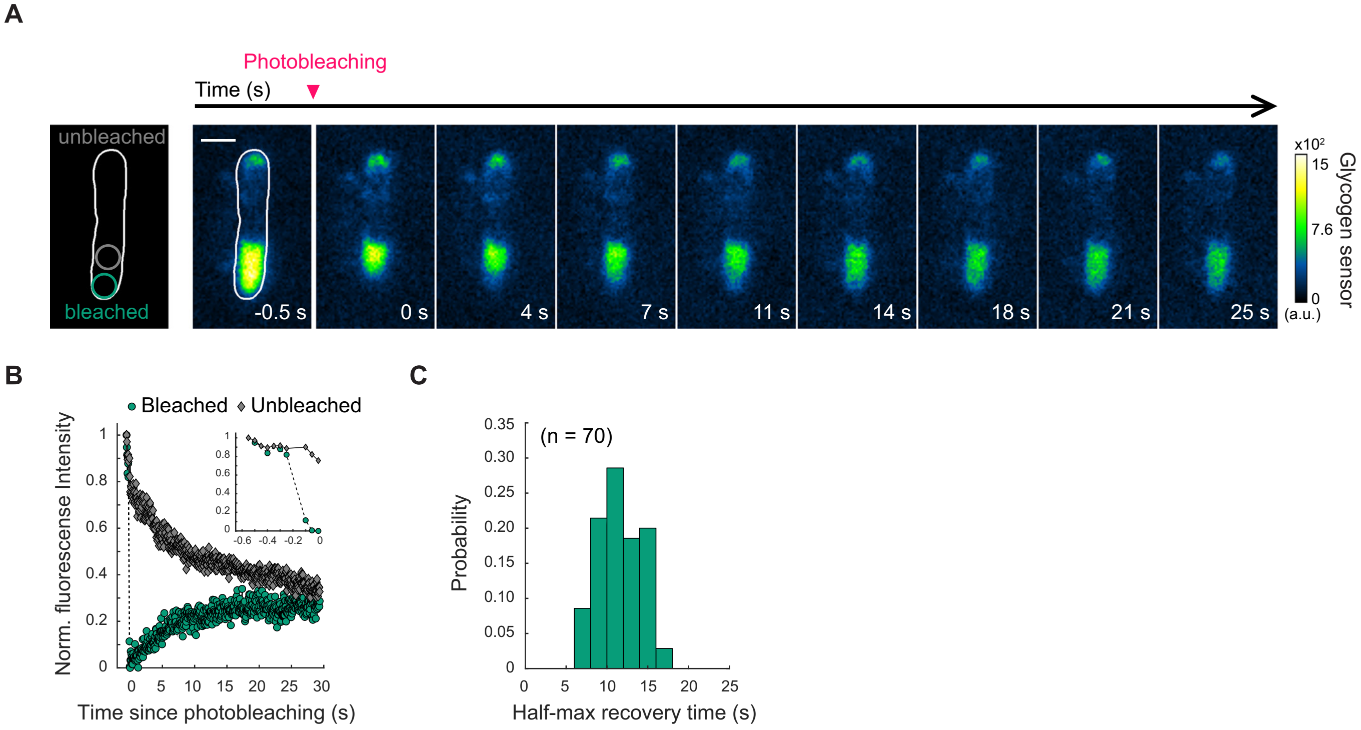

Figure EV8. FRAP measurements of the fluorescent glycogen sensor dynamics in glycogen-producing cells in transition phase.

(**A**) Time-lapse images of a glycogen-producing cell in transition phase before and after photobleaching a region of the cell pole with the larger glycogen sensor accumulation. The first image on the left shows the time frame before photobleaching, while the subsequent images represent frames captured after photobleaching at the indicated time. The schematic shows the region that was photobleached. Scale bar: 1 μm. (**B**) Plot showing the evolution of the normalized fluorescence intensity of the glycogen sensor for the unbleached region and the photobleached region of the cell shown in (**A**), before and after photobleaching. The inset shows the data before and during photobleaching. (**C**) Histogram of the half-max fluorescence recovery times calculated for 70 cells in which FRAP measurements were obtained. Source data are available online for this figure.

© The Author(s)

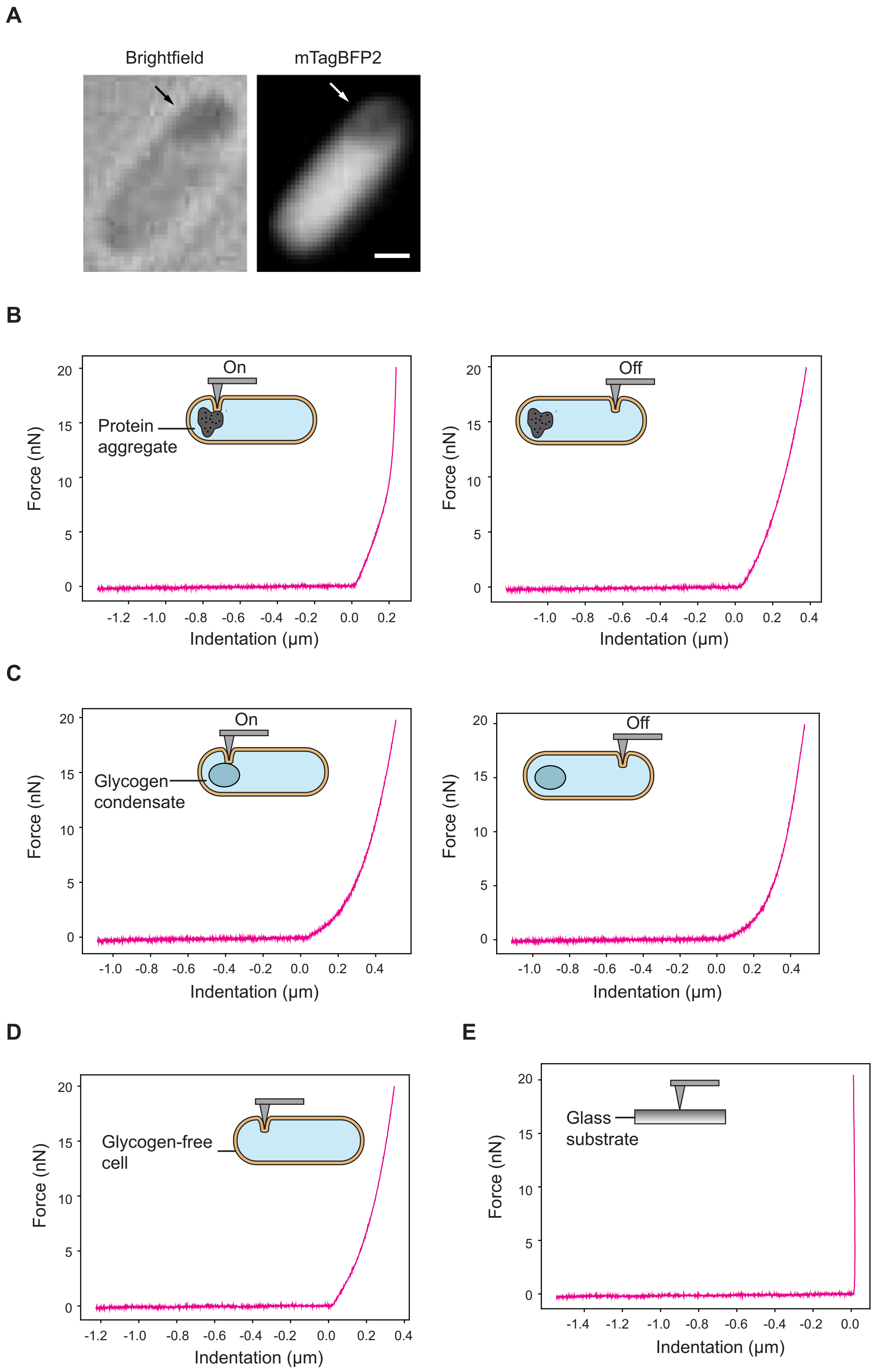
A
Brightfield
mTagBFP2
B
On
Protein aggregate
Off
Force (nN)
Indentation (µm)
C
On
Glycogen condensate
Off
Force (nN)
Indentation (µm)
D
Glycogen-free cell
Force (nN)
Indentation (µm)
E
Glass substrate
Force (nN)
Indentation (µm)

Figure EV9. Force-distance curve examples of indentation-based AFM experiments.

The experimental conditions and strains are the same as in Fig. 7A–C. (**A**) Brightfield image of the protein aggregate-containing cell illustrated in Fig. 7B. The accompanying fluorescence image, which is a duplication of the image in Fig. 7B, is shown for comparison. (**B**) Representative force-distance curves of a cell at regions "On" and "Off" the protein aggregates. (**C**) Same as (**A**) but for a cell with glycogen condensates. (**D**) Same as (**A**) but for a glycogen-free cell. (**E**) Representative force-distance curve on the glass substrate is shown as a control. Source data are available online for this figure.

© The Author(s)

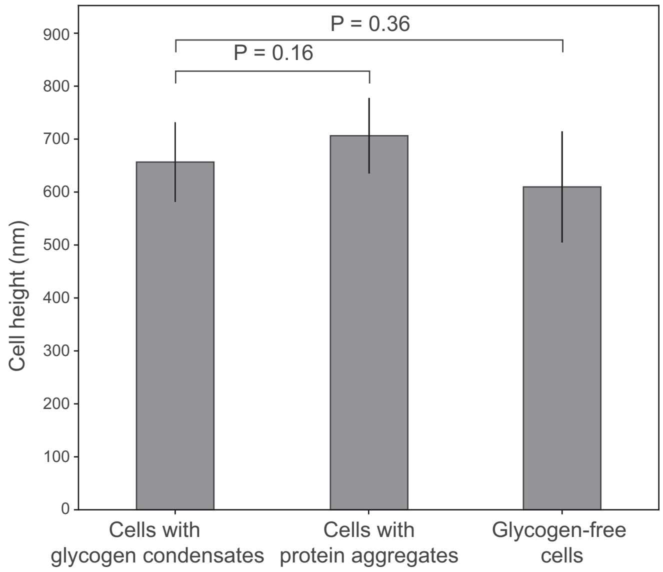

Figure EV10. Cell height measurements by AFM microscopy.

The experimental conditions and strains are the same as in Fig. 7A–C. Plot showing cell height measurements of 21 cells with protein aggregates, 7 cells with glycogen condensates, and 7 cells without glycogen. The cell height information was analyzed from AFM height images collected from more than three biological replicates. Displayed here are the mean values ± the standard deviations. Statistical comparisons were performed using an unpaired two-tailed Student's *t* test. Source data are available online for this figure.

© The Author(s)

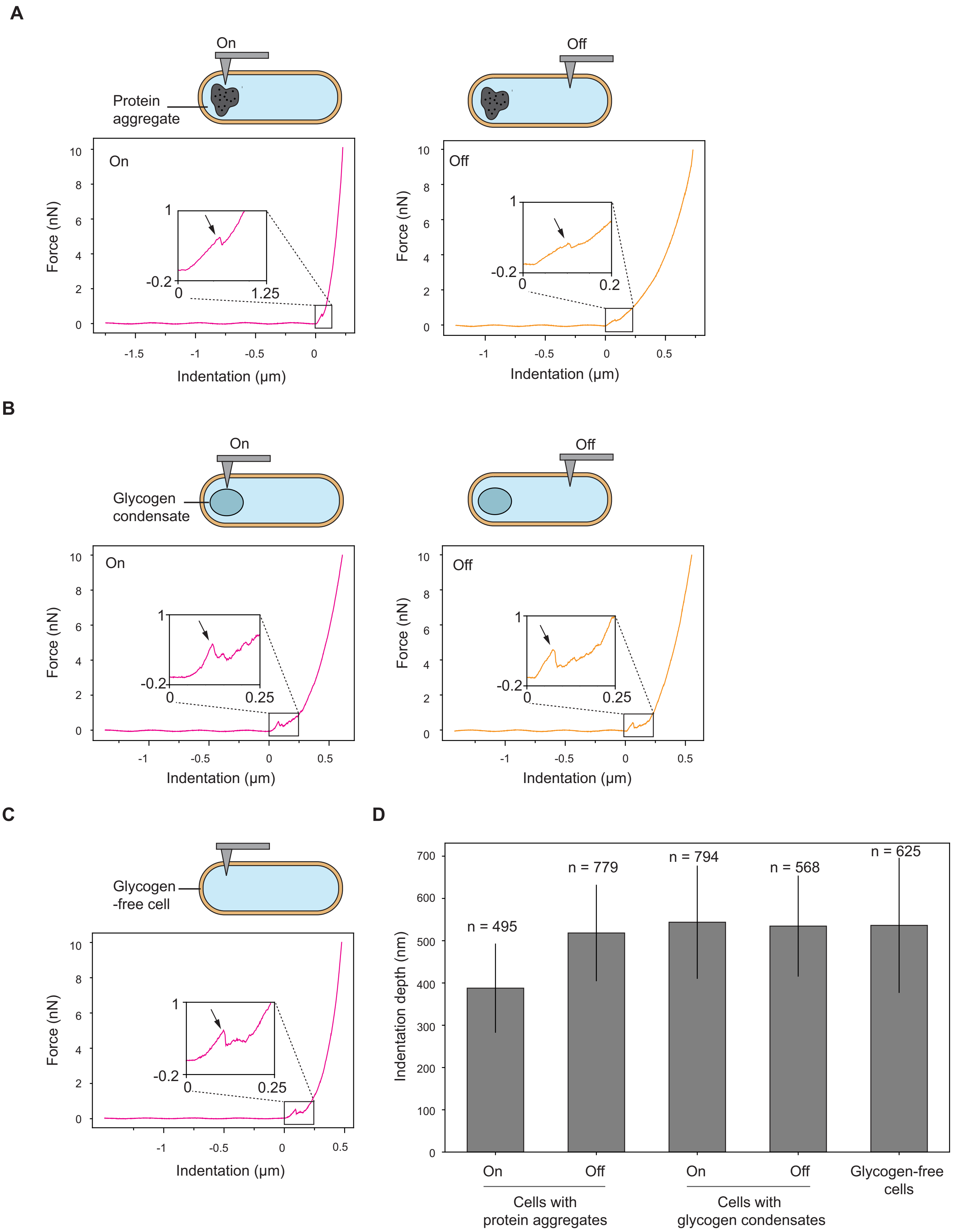
A
On
Protein aggregate
Off
On
Off
Force (nN)
Indentation (µm)
B
On
Glycogen condensate
Off
On
Off
Force (nN)
Indentation (µm)
C
Glycogen -free cell
Force (nN)
Indentation (µm)
D
Indentation depth (nm)
n = 495
n = 779
n = 794
n = 568
n = 625
On
Off
On
Off
Glycogen-free cells
Cells with protein aggregates
Cells with glycogen condensates

 © The Author(s)

Figure EV11. Force-distance curve examples of penetration-based experiments.

The experimental conditions and strains are the same as in Fig. 7D,E. Representative force-distance curves showing membrane puncture events (indicated by the arrow) in the figure inset. (**A**) Representative force-distance curves of a cell at regions “On” and “Off” the protein aggregates. (**B**) Same as (**A**) but for a cell with glycogen condensates. (**C**) Same as (**A**) but for a glycogen-free cell. (**D**) Plot showing the indentation depth distributions of 15 cells with protein aggregates, 16 cells with glycogen condensates, and 13 cells without glycogen. Shown are the mean values ± the standard deviations for the total number of indentation events (technical replicates) indicated by the *n* value. The indentation events were collected from more than three biological replicates. Source data are available online for this figure.
